# Effect of Immediate Thermal Exposure on the Surface Mechanical Performance of Polyurethane-Coated Oak Wood and Visible 3D-Printed Furniture Components

**DOI:** 10.3390/polym18172164

**Published:** 2026-09-04

**Authors:** Gabriela Slabejová, Jozef Fekiač, Lukáš Adamčík, Zuzana Vidholdová

**Affiliations:** 1 Department of Furniture and Wood Products, Faculty of Wood Sciences and Technology, Technical University in Zvolen, T. G. Masaryka 24, 96001 Zvolen, Slovakia; slabejova@tuzvo.sk (G.S.); jozef.fekiac@tuzvo.sk (J.F.); 2 Department of Woodworking, Faculty of Wood Sciences and Technology, Technical University in Zvolen, T. G. Masaryka 24, 96001 Zvolen, Slovakia; lukas.adamcik@tuzvo.sk; 3 Department of Wood Technology, Faculty of Wood Sciences and Technology, Technical University in Zvolen, T. G. Masaryka 24, 96001 Zvolen, Slovakia

**Keywords:** ABS-T, PET-G, PLA, polyurethane coating, surface mechanical properties, thermal exposure

## Abstract

A polyurethane coating represents the conventional solution for protecting and finishing visible wooden furniture surfaces. However, the increasing use of additive manufacturing has introduced visible 3D-printed polymer components whose surface mechanical performance is relevant to their application in furniture. This study investigated the effect of short-term exposure to 60 °C for 1 h on the surface mechanical properties of a pigmented polyurethane coating applied to oak wood and 3D-printed PLA, ABS-T and PET-G components. Specimens were evaluated under laboratory conditions (20 °C) and immediately after exposure to 60 °C, while their surfaces remained at an elevated temperature. Impact resistance, abrasion resistance, and scratch resistance using a tungsten carbide tip were determined according to the relevant standards, and the surface damage was assessed by visual inspection and digital microscopy. The polyurethane coating exhibited the smallest impact indentation diameter but showed earlier crack initiation and lower abrasion resistance than the 3D-printed polymers. Among the investigated polymers, ABS-T provided the most balanced combination of impact resistance, abrasion resistance and surface hardness, whereas PLA exhibited the greatest dimensional changes after exposure to 60 °C. Microscopic analysis revealed surface defects that were not detectable by visual inspection, demonstrating the value of digital microscopy for detecting subtle surface damage. The results indicate that ABS-T is a promising material for visible furniture components exposed to short-term elevated temperatures, while PET-G should be used with caution in applications exposed to radiant heat or direct sunlight.

## 1. Introduction

Additive manufacturing technologies have experienced significant development in recent years and are increasingly being adopted in various industrial sectors, including furniture production [1,2,3,4]. Among these technologies, fused deposition modelling (FDM) has become one of the most widely used additive manufacturing methods due to its relatively low cost, material availability, and capability to produce customized components with complex geometries [5]. In furniture manufacturing, FDM technology is no longer limited to rapid prototyping but is progressively being employed for the production of functional components, connectors, fittings, and decorative elements [6,7,8].

The growing interest in individualized furniture design and small-series production has increased the demand for customized joining systems that can be rapidly manufactured and adapted to specific construction requirements [9,10]. Advances in additive manufacturing have enabled the production of furniture connectors from various thermoplastic materials, particularly polylactic acid (PLA) and polyethylene terephthalate glycol-modified (PET-G) and acrylonitrile butadiene styrene-based materials (ABS) [11,12]. These materials differ considerably in their mechanical behaviour, thermal stability, and resistance to surface damage, which directly affects their suitability for furniture applications [13].

Recent trends in furniture design have increasingly employed visible joining elements as both structural and aesthetic components. Unlike conventional concealed fittings, modern connectors may intentionally remain exposed and become an integral part of the furniture appearance [14]. Examples include decorative spline joints and Hoffmann-type keys and customized connectors produced by additive manufacturing [15]. As visible components, these connectors are exposed to the same service conditions as adjacent furniture surfaces, including scratching, impact loading, local abrasion, and elevated temperatures resulting from nearby heat sources or direct solar radiation. Such conditions may occur, for example, in furniture placed close to south-facing windows, in glazed interiors with high solar gains, or in vehicle interiors, where surface temperatures may increase substantially during service.

In contemporary furniture manufacturing, polyurethane (PU) coating systems represent one of the most widely used surface finishing technologies [16]. Polyurethane coatings are valued for their balanced combination of hardness, scratch resistance, impact resistance, and long-term durability [17,18,19,20,21,22]. Due to these properties, PU-coated wood surfaces are commonly applied to furniture components subjected to everyday mechanical loading. As a result, polyurethane coating systems may be considered a benchmark representing the current standard of surface performance in furniture applications.

Although numerous studies have investigated the mechanical properties of 3D-printed polymers, most research has focused on tensile strength, flexural properties, layer adhesion, or dimensional stability [23,24,25,26,27,28]. In contrast, the surface mechanical performance of 3D-printed polymer components under exposure to elevated temperature remains insufficiently characterized. This issue is particularly relevant for visible furniture components, where surface integrity, appearance, and resistance to mechanical damage are important functional requirements. Moreover, comparative information on the surface response of 3D-printed polymer components and conventional PU-coated furniture surfaces under elevated temperature is scarce.

From the perspective of furniture engineering, such a comparison is particularly relevant because visible 3D-printed connectors are expected to withstand service conditions similar to those experienced by surrounding PU-coated furniture surfaces. However, differences in material structure between 3D-printed polymers and continuous polyurethane-coated surfaces may lead to different mechanisms of surface damage. Understanding their behaviour under elevated temperatures and mechanical loading may therefore facilitate material selection and the design of durable furniture components.

In practical furniture applications, elevated temperatures may act on components for relatively short periods without constituting conventional thermal treatment or long-term thermal ageing. For example, surfaces exposed to direct solar radiation or located near windows or other heat sources may reach elevated temperatures while simultaneously being subjected to mechanical contact or impact. The immediate mechanical response of polymer components under such conditions may differ from their behaviour after cooling to room temperature. Therefore, evaluating the material while still in its heated state can provide complementary information relevant to short-term service conditions that is not captured by conventional testing performed after thermal exposure and subsequent cooling.

The aim of this study was to evaluate the influence of immediate thermal exposure on the selected surface mechanical properties of a conventional PU coating and visible 3D-printed polymer components manufactured from PLA, ABS-T, and PET-G. The investigated properties included impact resistance, abrasion resistance, and surface hardness, which were evaluated immediately after thermal exposure while the specimen surfaces remained at an elevated temperature. Particular attention was devoted to microscopic assessment of surface damage to identify cracks and scratches not detectable by conventional visual evaluation. Such defects may adversely affect not only the aesthetic appearance but also the durability and service life of furniture components.

The novelty of the study lies in the direct comparison of three 3D-printed polymer materials with a conventional PU-coated furniture surface under immediate thermal exposure, combined with microscopic characterization of the resulting surface damage. The microscopic analysis further revealed cracks frequently occurring along the interfaces between adjacent deposited filaments, suggesting that these interfaces may represent preferential paths for crack propagation under the investigated conditions.

## 2. Materials and Methods

The study investigated the effect of surface finish and thermal exposure on selected surface mechanical properties. The experimental factors and response variables are summarized in Table 1.

### 2.1. Materials and Surface Finish

**Figure 1:**
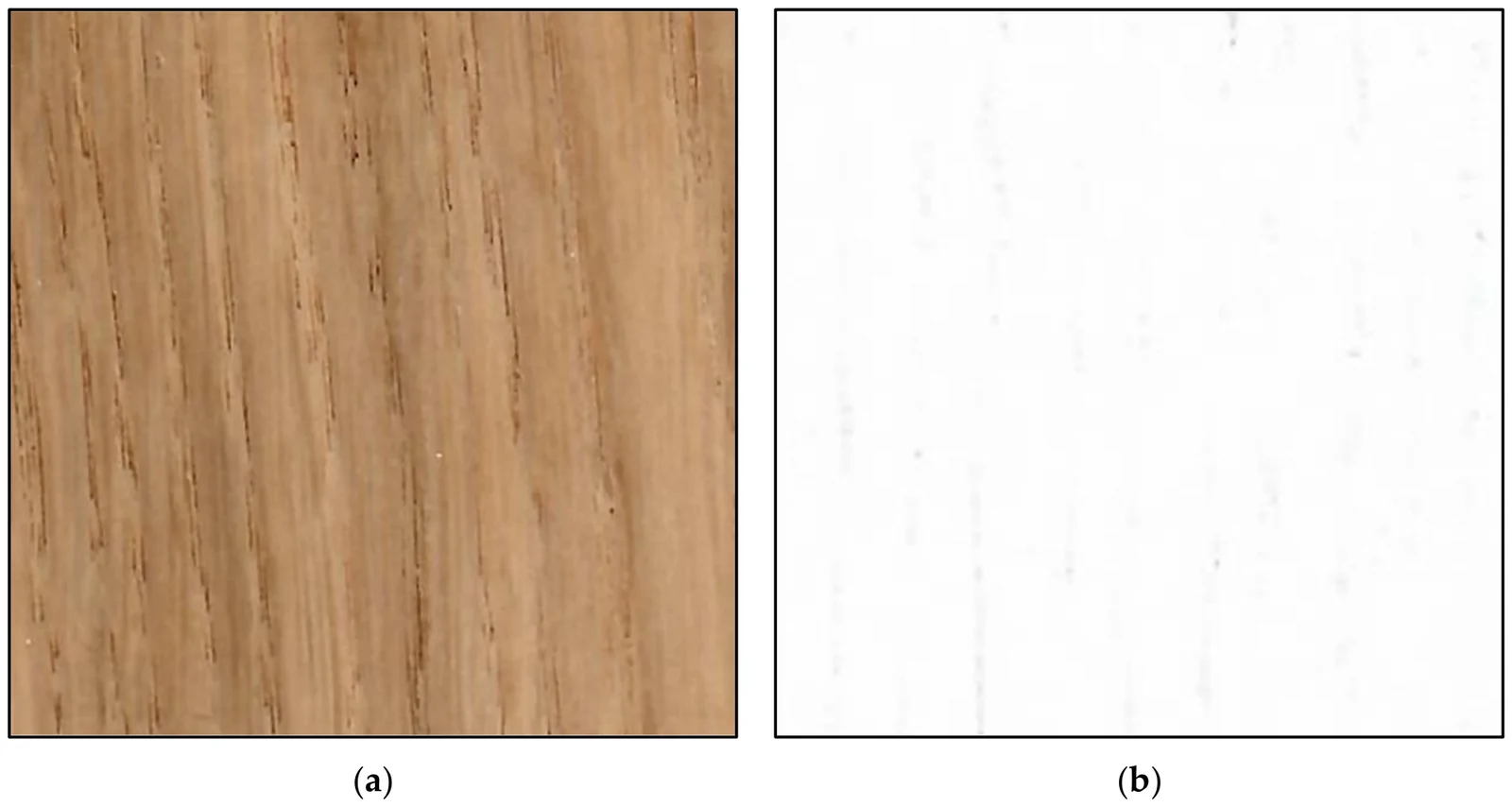
Oak substrate and polyurethane coating used in the study: (**a**) European oak (*Quercus robur* L.) substrate; (**b**) polyurethane-coated specimen.

European oak (*Quercus robur* L.) wood was used as the substrate material (Figure 1a). Test specimens were prepared from radial-tangential boards with dimensions of 20 mm × 200 mm × 400 mm in the impact resistance, pencil hardness and tungsten carbide tip scratch test. Test specimens were prepared with dimensions of 20 mm × 100 mm × 100 mm in the abrasion resistance test. The specimens’ surfaces were sanded using an eccentric sander (DeWalt, Towson, MD, USA) with an oscillation diameter of 2.6 mm and finished with P120 abrasive paper.

A pigmented polyurethane coating material (6OPP530NI, SIRCA S.p.A., Massanzago, Italy) [33] intended for interior applications (Figure 1b) was applied according to the manufacturer’s recommendations (Table 2).

The polyurethane surface system consisted of two coats, resulting in an average dry film thickness of 85 ± 5 µm.

Three commercially available polymer filaments supplied by Filament PM (Haňovice, Czech Republic) [34] were selected for the production of 3D-printed fasteners: polylactide (PLA), acrylonitrile–butadiene–styrene modified with methyl methacrylate (ABS-T), and glycol-modified polyethylene terephthalate (PET-G). These filaments were selected due to their widespread use in additive manufacturing and their different thermal resistance characteristics. According to the manufacturer, the maximum service temperatures are approximately 60–70 °C for PLA, 98 °C for ABS-T, and 75–85 °C for PET-G.

**Figure 2:**
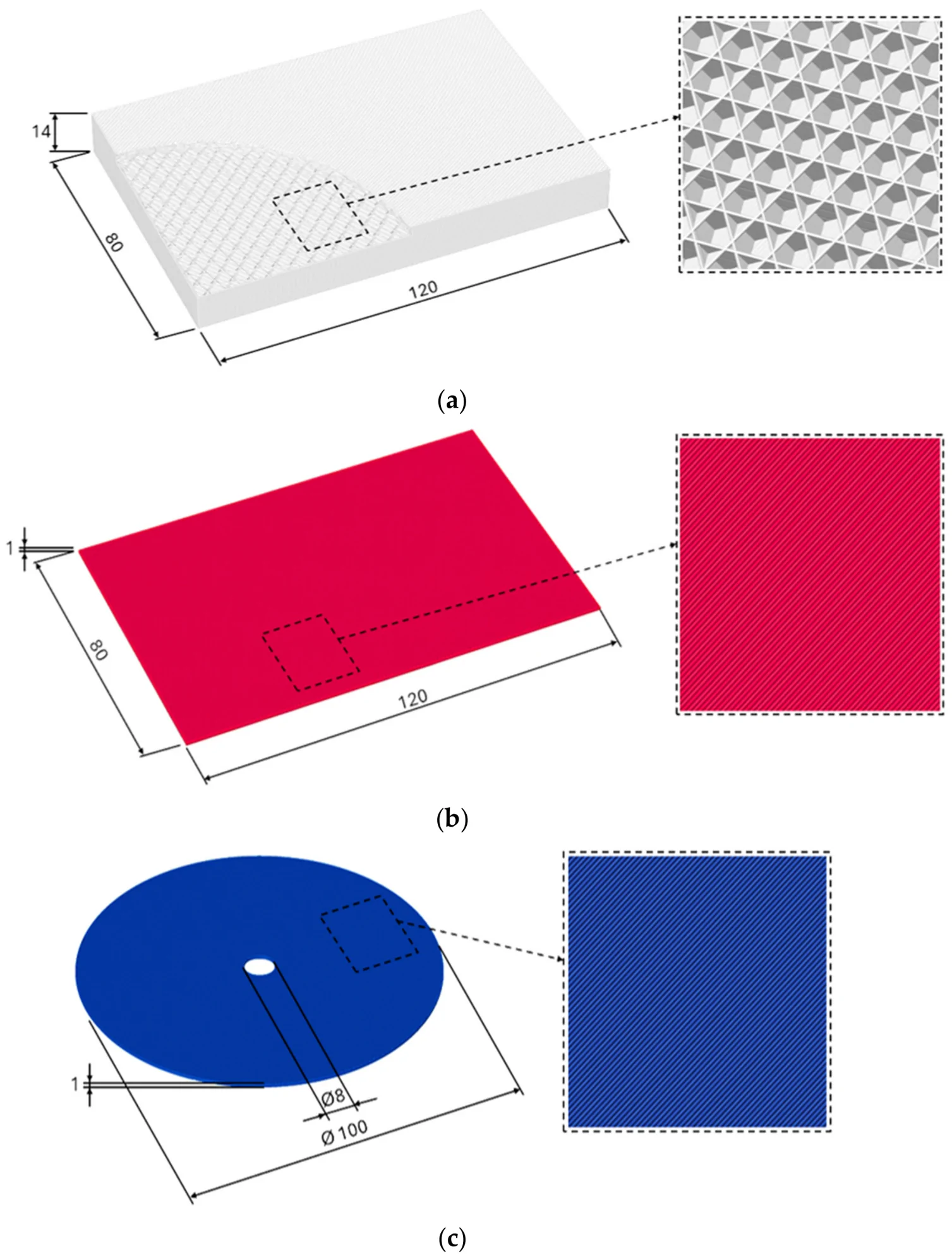
Dimensions (mm) and geometries of specimen: (**a**) specimen I with an internal cubic structure; (**b**) specimen II printed without infill using a monotonic layer pattern; and (**c**) specimen III printed without infill using a monotonic layer pattern.

Three specimen types were prepared: Type I specimens (80 mm × 120 mm × 14 mm) with an internal cubic structure, three top surface layers, and three bottom surface layers (Figure 2a, Table 3) for impact resistance testing; Type II specimens (80 mm × 120 mm × 1 mm) consisting of five printed layers (Figure 2b, Table 3) for hardness and scratch-resistance testing; and Type III specimens (Ø 100 mm × 1 mm) consisting of five printed layers (Figure 2c, Table 3) for abrasion resistance testing.

The specimens were printed on a Bambu Lab X1-Carbon 3D printer. Individual filaments were dried under different conditions (Table 3) directly in the filament dryer, which is part of the 3D printer. After drying, the maximum moisture content of the material was w = 5% according to the recommendations in the technical data sheets.

### 2.2. Thermal Exposure

All specimens were conditioned under laboratory conditions (20 ± 2 °C and 50 ± 5% relative humidity (RH)) for 14 days prior to testing. Subsequently, both the PU-coated oak specimens and the 3D-printed polymer specimens were divided into two experimental groups. The first group was tested under laboratory conditions (20 ± 2 °C and 50 ± 5% RH). The second group was exposed to an elevated temperature of 60 ± 2 °C in an LSN11/ST laboratory dryer with forced-air circulation (SnolTherm, Narkūnai, Lithuania) for 1 h.

The exposure temperature of 60 °C was selected as an elevated but sub-melting temperature relevant to the intended application of the investigated materials. According to the thermal analysis presented in study [35], the main melting transition of PLA occurred at approximately 150–160 °C; therefore, the selected temperature remained well below the melting range while providing a sufficiently elevated condition to induce changes in the surface mechanical behaviour. An exposure time of 60 min was selected to allow the specimens to reach and equilibrate at the target temperature before the subsequent mechanical evaluations.

Immediately after removal from the dryer, the surface temperature of each specimen was measured using an Almemo 26908A instrument (Ahlborn Mess- und Regelungstechnik GmbH, Holzkirchen, Germany). The specimens were then immediately subjected to the evaluation of their surface mechanical properties while still at the elevated temperature. To minimize specimen cooling prior to testing, the impact and abrasion tests were initiated immediately after the surface temperature measurements. Following completion of the mechanical tests, all specimens were conditioned under laboratory conditions (20 ± 2 °C and 50 ± 5% RH) for 24 h, after which their dimensions were measured using a digital calliper.

### 2.3. Surface Performance Evaluation

The surface mechanical performance of the 3D-printed polymer specimens was evaluated in comparison with a conventional polyurethane coating, representing the standard surface finishing system used in the furniture industry. The evaluated properties included impact resistance, abrasion resistance, and surface hardness.

#### 2.3.1. Impact Resistance

The impact resistance was determined according to the standard STN EN ISO 6272-2 [29], using a falling-weight tester equipped with a small-area indenter. The specimens were tested at drop heights of 10, 25, 50, 100, 200 and 400 mm. The diameter of the indentation was measured and the surface damage was visually assessed according to the classification presented in Table 4. Six independent measurements were performed (n = 6).

#### 2.3.2. Abrasion Resistance

Abrasion resistance was evaluated according to STN EN ISO 7784-1 [30]. The abrasion test was performed for 100 revolutions in accordance with the standard. Six independent measurements were performed (n = 6). The abrasion resistance coefficient *K_T_* was calculated as the difference between the specimen mass before (m_1_) and after (m_2_) the abrasion test:

K_T_ = (m_1_ − m_2_), (1)

where m_1_—Specimen weight before sanding (g), m_2_—Specimen weight after sanding (g).

#### 2.3.3. Surface Hardness

Surface hardness was assessed using two complementary methods.

Pencil hardness was determined according to STN EN ISO 15184 [31]. The test was performed from the softest to the hardest pencil grade, and the hardness was defined by the first pencil producing a visible scratch on the specimen surface (Table 5). Three independent measurements were performed (n = 3).

Scratch resistance was further evaluated using a tungsten carbide tip according to STN EN 438-2+A1 [32]. The tip was drawn across the specimen surface under progressively increasing normal loads (1–8 N). Surface damage was visually inspected at a 45° viewing angle from a distance of approximately 30–40 cm. Three independent measurements were performed (n = 3). The surface hardness corresponded to the lowest applied load that produced a visible scratch.

### 2.4. Microscopic Analysis

Following the mechanical tests, the damaged surfaces were visually examined using a tabletop magnifier and subsequently analyzed with a VHX-7000 digital microscope (Keyence Corporation, Osaka, Japan). Although microscopic examination is not required by the applied testing standards, it was employed to provide a more detailed characterization and documentation of the surface damage mechanisms. The microscopic observations complemented the standard visual assessment and enabled a more accurate comparison of the damage morphology of the polyurethane coating and the 3D-printed polymer specimens.

The criteria for microscopic damage classification were based on the type of mechanical test. In the impact test, indentations visible to the naked eye or with a magnifying glass were subsequently examined microscopically to identify and confirm the presence of cracks. For the pencil- and tip-scratch tests, scratches visible to the naked eye or with a magnifying glass were examined microscopically to assess disruption of the surface structure. Scratches that were macroscopically apparent primarily as a change in surface gloss were considered surface damage when microscopic examination confirmed disruption of the surface structure.

### 2.5. Statistical Analysis

The experimental data were analyzed using Microsoft Excel for Microsoft 365 and STATISTICA 14 software. Descriptive statistics (mean ± standard deviation) were calculated for all measured variables. Statistical significance was set at *p* < 0.05. A three-way analysis of variance (ANOVA) was used to evaluate the effects of material type, thermal exposure, and drop height on impact resistance (indentation diameter), while a two-way ANOVA was applied to assess the effects of material type and thermal exposure on the abrasion resistance coefficient (K_T_). Both the main effects and interactions between the investigated factors were evaluated. Additionally, 95% confidence intervals were calculated for the mean values of impact resistance and abrasion resistance in each experimental group. Prior to performing the ANOVA, the assumptions of normality of residuals and homogeneity of variances were verified.

## 3. Results and Discussion

Since thermal exposure may induce permanent dimensional changes in polymeric materials, the dimensions of the 3D-printed polymer specimens were measured before thermal exposure and after subsequent mechanical testing and conditioning under laboratory conditions. The results, summarized in Table 6, were used to evaluate the permanent dimensional stability of the investigated materials.

**Figure 3:**
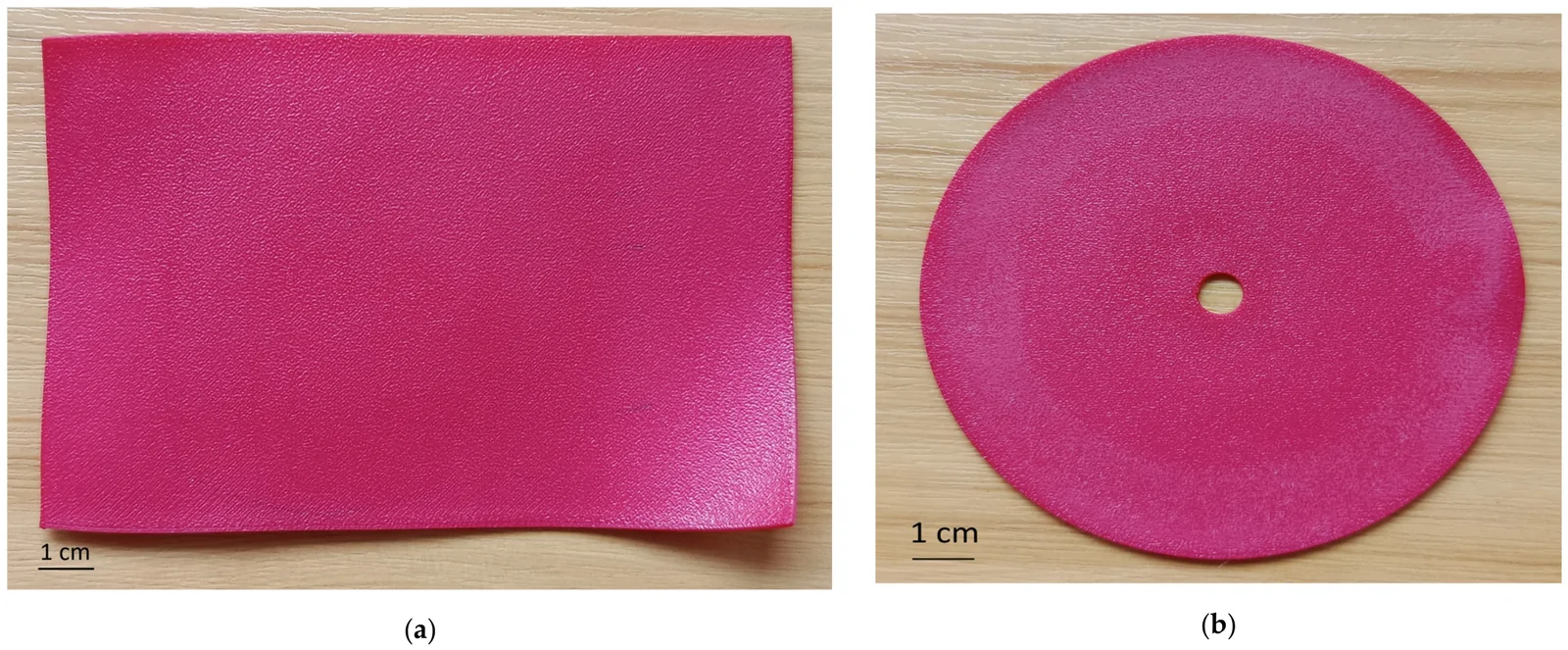
Deformation of PLA specimens after exposure to 60 °C: (**a**) specimen II and (**b**) specimen III.

Shbanah et al. [36] report that the size of the PLA test specimens was controlled by using a calliper, so there was not a measurable difference before and after the heat treatment in the case of the treatments at 55 °C, 65 °C and 80 °C. They concluded that the heat treatment at 95 °C caused a measurable difference in the dimensions of the test specimens’ PLA and a deformation. The measurements in this study refute this claim, because even at a temperature of 60 °C, there was a significant change in the dimensions of the PLA test specimens and deformation specimen II (Figure 3a) and specimen III (Figure 3b). Test specimens made of oak wood with a polyurethane coating and of ABS-T and PET-G could be considered stable at 60 °C. They showed no significant dimensional change (Table 6) or deformation. Seyedzavvar and Boğa [37] show in their paper that ABS is favoured for functional prototypes and engineering applications because of its moderate tensile strength and thermal resistance.

The observed dimensional response may be related to the different thermal behaviour of the investigated polymers and their sensitivity to temperature-induced stress relaxation. The absence of measurable deformation in ABS-T and PET-G indicates that these materials maintained their dimensional stability under the applied short-term thermal exposure. In contrast, the dimensional changes observed for PLA suggest that its use in visible furniture components should be considered carefully where temporary exposure to elevated temperatures is expected.

These findings suggest that 3D-printed components should be evaluated under the actual temperature conditions in which they may be used, rather than solely after cooling.

### 3.1. Impact Resistance and Abrasion Resistance

Impact resistance was evaluated based on visual assessment of surface damage using both a tabletop magnifier and a digital microscope, together with measurements of the indentation diameter after impact. Table 7 summarizes the classification of surface damage observed on the polyurethane coating and polymer surfaces. Two testing conditions were evaluated: specimens tested under laboratory conditions (20 ± 2 °C) and specimens tested immediately after thermal exposure at 60 ± 2 °C, while their surfaces were still at an elevated temperature.

Detailed microscopic examination of the impacted surfaces provided additional information on the damage mechanisms. The microcracks were detectable during conventional visual inspection or at 10× magnification, but became clearly visible under digital microscopy at magnifications between 100× and 300×. This observation is important not only from an aesthetic perspective but also for assessing the surface structural integrity, and long-term durability of an element made of polymer filament. Whereas damage to the polyurethane coating represents primarily an aesthetic defect, cracking of a visible 3D-printed fastener may compromise the structural integrity of surface and serviceability of the furniture joint.

**Figure 4:**
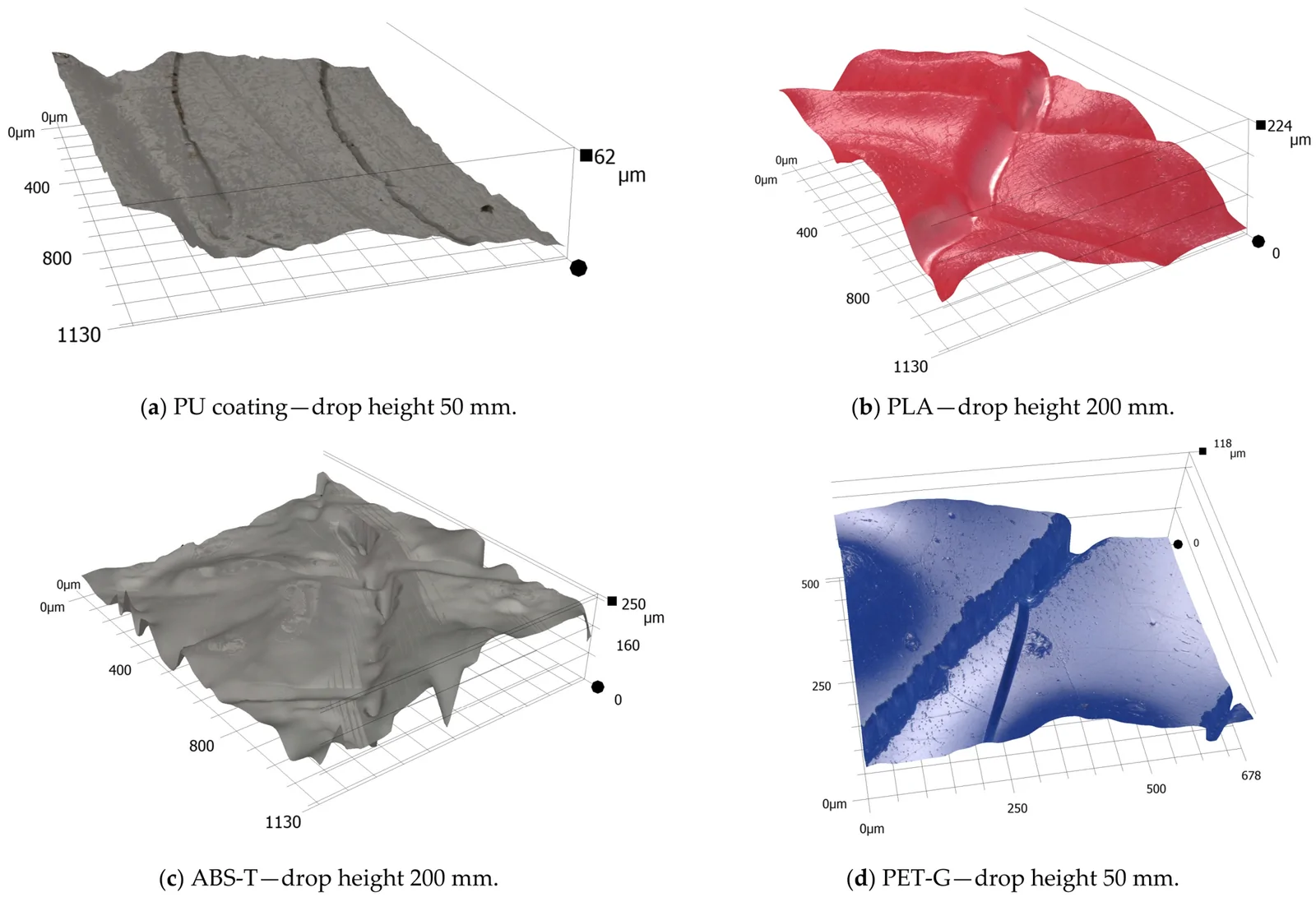
Representative impact damage of the tested materials immediately following exposure to 60 °C: (**a**) polyurethane coating; (**b**) PLA; (**c**) ABS-T; and (**d**) PET-G.

The polyurethane coating exhibited the first visible cracks after impact from a drop height of 50 mm under both testing conditions (Figure 4a). The formation of cracks in a polyurethane coating after a rapid-deformation test is reported in the paper [38,39,40]. In our earlier work [40], microcracks were detected at even lower drop heights, namely 25 mm for glossy coatings and 10 mm for matte coatings, indicating that coating formulation and surface finish substantially influence crack initiation. Shaily et al. [39] reported that the decomposition of the urethane group, which occurs below 300 °C, is the first degradation step in the thermal decomposition of polyurethane. Since the exposure to temperature applied in the present study (60 °C) was considerably lower, no thermal degradation of the polyurethane coating was expected, and the observed damage can therefore be attributed primarily to mechanical loading.

Thermal exposure affected the impact behaviour of the investigated polymer materials differently. In PLA specimens, the indentation diameter produced by a 400 mm drop height decreased significantly following elevated-temperature exposure, while microcracks were first detected after impact from 200 mm (Figure 4b). In contrast, non-exposed PLA specimens exhibited microcracks already at a drop height of 100 mm, indicating improved impact resistance immediately following exposure to 60 °C.

A different behaviour was observed for ABS-T and PET-G, in which thermal exposure reduced impact resistance. In ABS-T, cracks appeared during thermal exposure at a drop height of 200 mm, whereas non-exposed specimens exhibited cracking only after impact from 400 mm (Figure 4c). This behaviour is consistent with the fracture characteristics of ABS reported by Seyedzavvar and Boğa [37] and En-naji et al. [41], who described ABS as a relatively rigid and notch-sensitive material exhibiting limited plastic deformation and rapid crack propagation under impact loading.

The greatest reduction in impact resistance during thermal exposure was observed for PET-G. Microcracks appeared after impact from a drop height of only 50 mm following thermal exposure, whereas specimens tested under laboratory conditions showed cracking at 100 mm (Figure 4d). Although PLA generally exhibits higher mechanical strength than PET-G, PET-G is usually characterized by lower thermal stability [42]. The present results therefore indicate that thermal exposure increased the brittleness of PET-G to a greater extent than PLA, while ABS-T retained the highest impact resistance among the investigated polymers. Microscopic observations further revealed that the cracks partially followed the deposition pattern of adjacent printed filaments, suggesting that the interfaces between neighbouring extruded roads could represent preferential paths for crack propagation under impact loading.

The observed differences can be related to the distinct thermal sensitivity of the investigated polymers and their response to loading while still at an elevated temperature. At 60 °C, the polymer matrix may exhibit increased molecular mobility and reduced resistance to localized deformation, although the extent of this effect depends on the polymer type and its thermal characteristics. The improved impact response of PLA may be associated with short-term stress relaxation during heating, whereas the earlier crack initiation observed in ABS-T and PET-G suggests a greater reduction in resistance to impact loading in the heated state. Thus, the results primarily describe the immediate mechanical response of the materials at elevated temperature rather than permanent changes caused by thermal ageing.

These observations are supported by previous studies. Mishra et al. [43] associated the lower tensile strength of PET-G with brittle fracture behaviour, rapid crack propagation, and limited plastic deformation. Similarly, Castanon-Jano et al. [44] reported tensile strengths of approximately 60 MPa for PLA and 30 MPa for PET-G, confirming the superior mechanical performance of PLA. Shbanah et al. [36] demonstrated that heat treatment at 65 °C for 5 h increased the tensile strength of PLA by approximately 35% without causing dimensional deformation, which was attributed to stress relaxation and reduced structural porosity. A similar mechanism may contribute to the improved impact resistance of PLA observed in the present study, where crack initiation occurred only at higher impact energies during thermal exposure. Comparable conclusions regarding stress relaxation induced by thermal treatment have also been reported by other authors [36,45,46]. Seyedzavvar and Boğa [37] and Holcomb et al. [47] reported that PET-G may exhibit greater ductility and toughness than ABS, aided by its molecular structure, as ester linkages provide strength while glycol modifications reduce crystallinity and improve chain mobility, preventing brittle fracture The surface properties of 3D-printed polymer filaments are affected not only by temperature during indoor ageing, but also by other environmental factors, such as UV radiation. Tomáštík et al. [48] investigated the photodegradation of PLA, ABS-T, and PET-G and reported that PLA showed negligible changes in its mechanical and structural properties. In contrast, ABS-T exhibited increased surface hardness associated with higher brittleness and a greater tendency for microcrack formation. The most pronounced degradation was observed in PET-G, where changes in local mechanical properties were confined to a surface layer less than 10 μm deep. These findings are consistent with the greater susceptibility of PET-G to environmental degradation reported in previous studies.

Table 8 presents the ANOVA results for the impact resistance and the analysis of variance for the abrasion resistance under thermal exposure.

**Figure 5:**
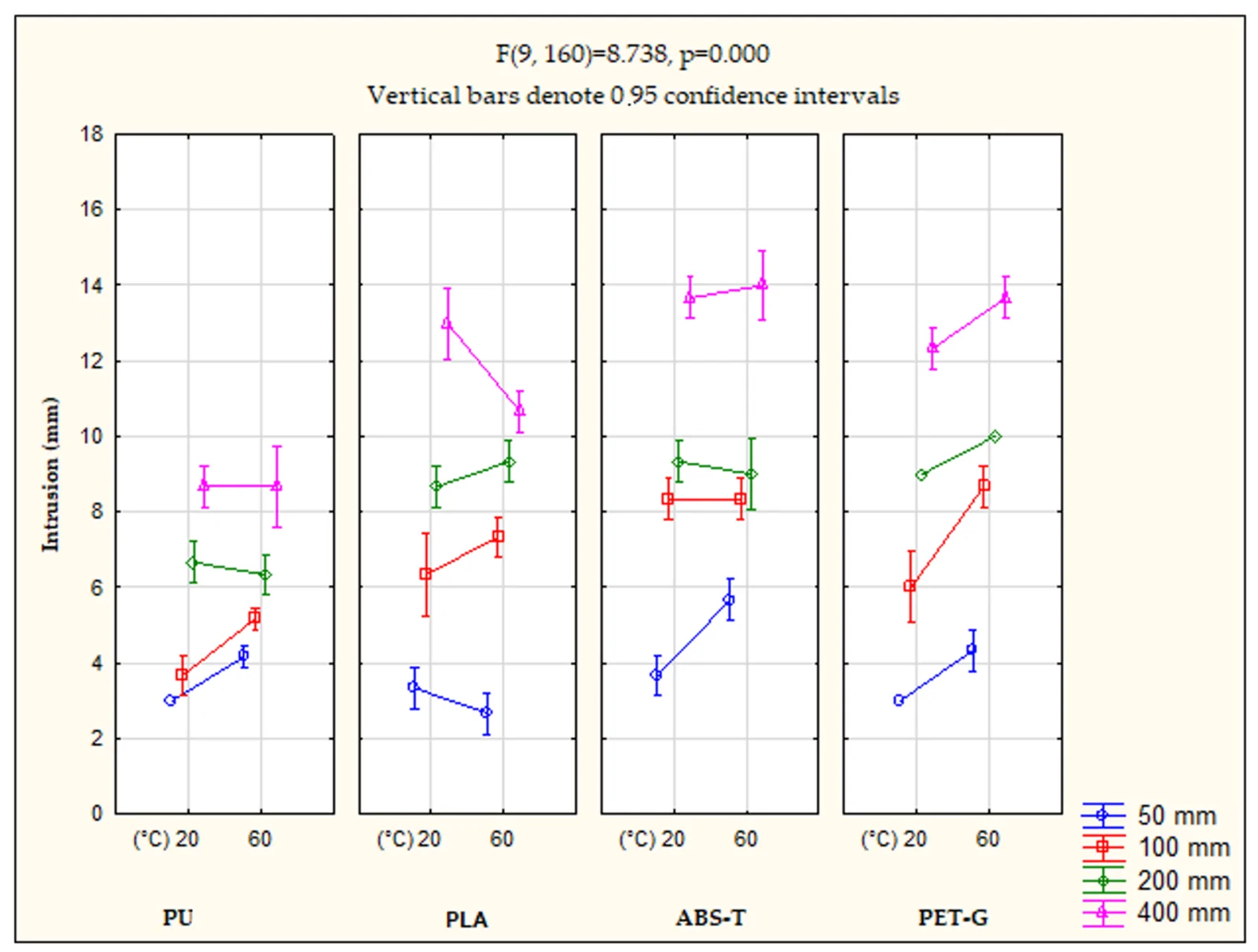
Mean indentation diameter with 95% confidence intervals after impact testing at drop heights of 50, 100, 200, and 400 mm for the polyurethane coating and 3D-printed PLA, ABS-T, and PET-G specimens before thermal exposure (20 °C) and during exposure to 60 °C for 1 h.

The three-way ANOVA revealed that material type, thermal exposure, and drop height all had a statistically significant effect on the indentation diameter measured after impact testing with a falling-weight tester equipped with a small-area indenter (Table 8). The surface temperatures of the thermally exposed specimens were measured immediately after removal from the laboratory dryer (Table 6), confirming that the impact tests were performed under elevated-temperature conditions. Significant differences between the polyurethane coating and the 3D-printed polymer materials were observed at drop heights of 100 mm and above (Figure 5). At the maximum drop height (400 mm), the polyurethane coating exhibited a significantly smaller indentation diameter than all investigated polymer materials under both laboratory and elevated-temperature conditions.

According to Kutsal et al. [49], filament type is the dominant factor affecting the mechanical performance of 3D-printed specimens, contributing 83.64% to tensile strength, 54.95% to elongation, and 88.51% to the modulus of elasticity. Similarly, the present study confirmed that the material (filament type) had a statistically significant effect on both the impact and abrasion resistance.

Shbanah et al. [36] reported that heat treatment is an effective approach for improving the mechanical properties of PLA after the 3D-printing process by reducing internal porosity and residual stresses. Although the objective of the present study was different, the impact resistance results showed a similar trend. The thermally exposed PLA specimens exhibited a smaller indentation diameter and delayed crack initiation than the non-exposed specimens, particularly at the highest drop height (400 mm), indicating improved resistance to localized impact loading.

Unlike the study by Shbanah et al. [36], the specimens in the present work were tested immediately after thermal exposure at 60 °C. Because the impact test was performed sequentially with increasing drop height, the heated specimens gradually cooled during the test. Therefore, the observed improvement in PLA impact resistance may be attributed to the combined effect of short-term thermal exposure and the progressive decrease in specimen temperature during testing.

**Figure 6:**
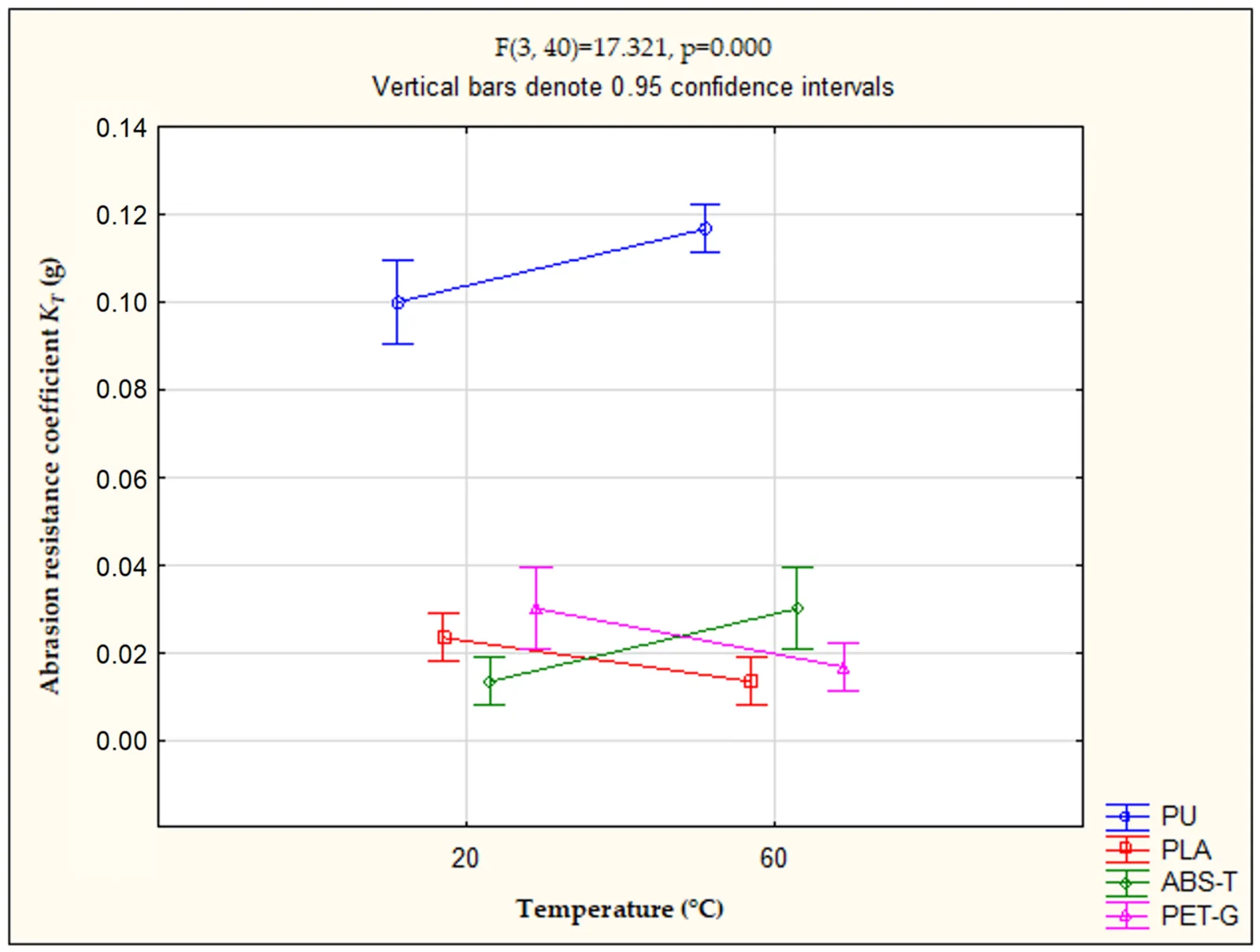
Mean abrasion resistance coefficient (K_T_ (g)) with 95% confidence intervals for the polyurethane coating and 3D-printed PLA, ABS-T, and PET-G specimens before thermal exposure (20 °C) and during exposure to 60 °C for 1 h.

The two-way ANOVA revealed that both material type and thermal exposure had a statistically significant effect on the abrasion resistance coefficient (*K_T_*), whereas their interaction was not statistically significant (Table 8). Similar findings were reported by Suresha et al. [50], who identified the filament type as the dominant factor affecting the abrasion resistance of 3D-printed polymers. Significant differences were observed between the polyurethane coating and all investigated 3D-printed polymer materials, both under laboratory and elevated-temperature conditions (Figure 6).

The polyurethane coating exhibited the greatest material loss during abrasion testing, whereas all 3D-printed polymer materials showed significantly lower material loss. Yang et al. [51] demonstrated that incorporating hard micro- and nanoscale particles into a polyurethane-based coating can improve its mechanical robustness and resistance to surface wear, highlighting the importance of coating composition and surface structure in determining the mechanical durability of polyurethane coatings. In our previous study [40], the abrasion loss of a pigmented polyurethane coating reached up to 0.5 g, whereas the polyurethane coating investigated in the present study exhibited substantially higher abrasion resistance. Among the investigated polymers, ABS-T showed the highest abrasion resistance, while PET-G exhibited the lowest under laboratory conditions (20 °C) (Figure 6).

These findings differ from those reported by Kujawa and Ptak [52], who evaluated abrasion resistance based on volumetric material loss and concluded that PET-G exhibited the highest abrasion resistance, whereas ABS showed the lowest. The discrepancy may be attributed to differences in the investigated ABS material, as the ABS-T filament used in the present study was modified with methyl methacrylate, which may have enhanced its surface resistance to abrasion. Differences in printing parameters and testing methodology may also have contributed to the observed variation between the studies.

The effect of exposure to 60 °C on abrasion resistance depended on the polymer type. While abrasion loss increased for the polyurethane coating and ABS-T, it decreased for PLA and PET-G, indicating improved abrasion resistance under the applied test conditions.

### 3.2. Pencil Hardness and Scratch Resistance

The surface hardness of the polyurethane coating and the 3D-printed polymers specimens determined by the pencil test and tungsten carbide tip test is shown in Table 9.

**Figure 7:**
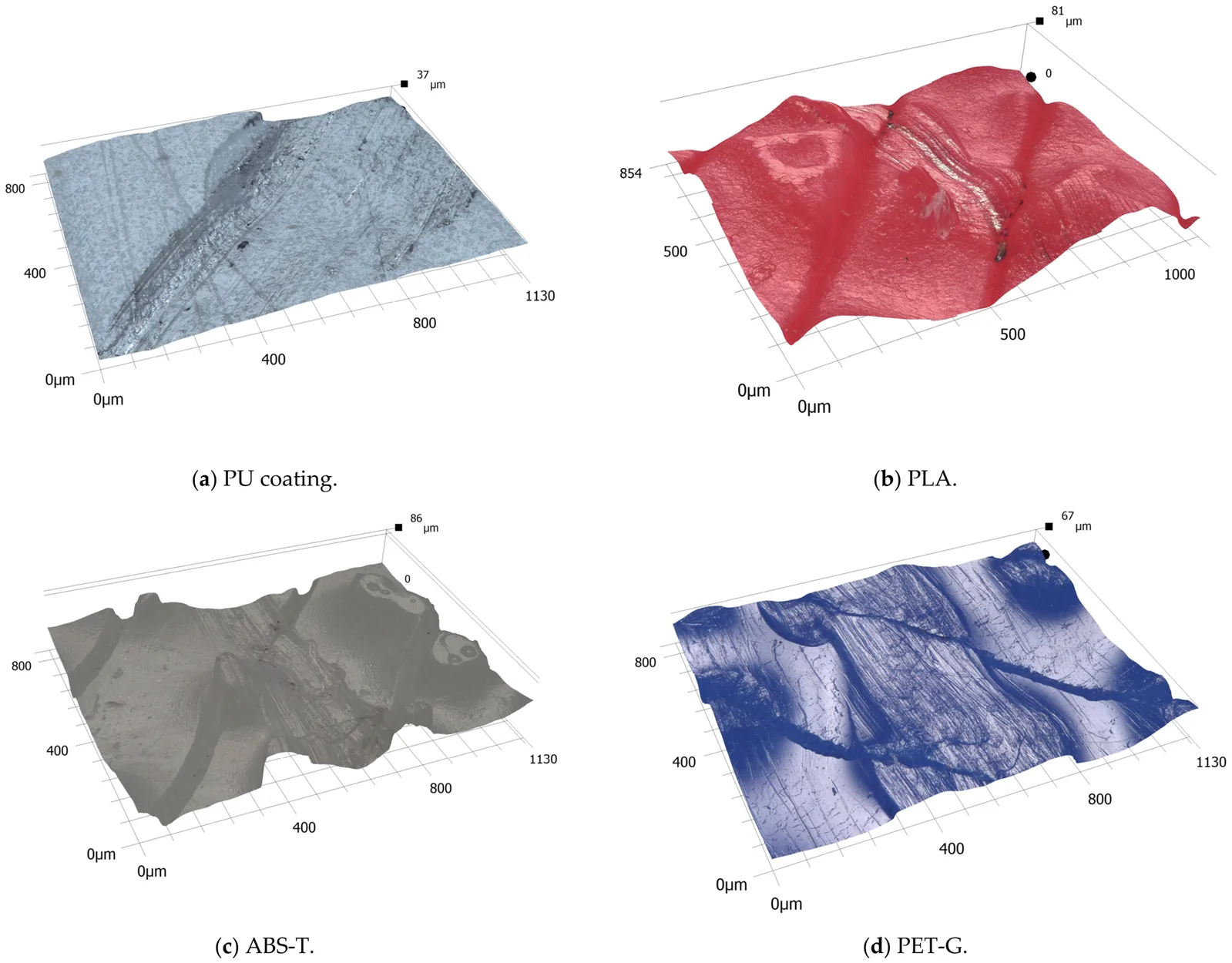
Representative damage of the tested materials immediately following exposure to 60 °C, evaluated using the pencil hardness test: (**a**) polyurethane coating; (**b**) PLA; (**c**) ABS-T; and (**d**) PET-G.

Under laboratory conditions, the polyurethane coating reached a pencil hardness of grade 3H. When tested immediately after thermal exposure to 60 °C, the hardness decreased by one grade to H (Figure 7a). Similar hardness values for polyurethane coatings have been reported in the literature. Shaily et al. [39] found pencil hardness values ranging from HB to H for coatings cured at room temperature and 3H to 4H for coatings cured at elevated temperature. Likewise, our previous study [40] reported pencil hardness values ranging from F to 3H, which is in good agreement with the present results. Unlike these studies, however, the specimens in the present work were tested immediately after exposure to 60 °C, while the coating surface was still at an elevated temperature. Therefore, the measured decrease in hardness reflects the immediate mechanical response of the coating rather than changes associated with complete cooling or post-curing.

Compared with the polyurethane coating, ABS-T exhibited the highest pencil hardness under laboratory conditions (grade 8; 4H), whereas PLA and PET-G reached grade 6 (H). Immediately after thermal exposure, the hardness decreased by one grade for PLA (H → F) and by two grades for ABS-T (4H → H), while PET-G showed no measurable change (Figure 7d). The unchanged hardness of PET-G agrees with the thermal behaviour reported by Mishra et al. [43], who determined a glass transition temperature of approximately 80.9 °C, indicating that exposure to 60 °C is insufficient to induce substantial structural changes. In contrast, the decrease observed for PLA and ABS-T indicates a temporary reduction in surface resistance at elevated temperature.

In the pencil test, damage was defined as a scratch remaining visible after removal of the graphite trace. On the 3D-printed polymer specimens, the scratch was often manifested only by a slight change in surface gloss. However, microscopic examination confirmed that these apparently minor defects corresponded to actual penetration of the stylus into the material (Figure 7).

**Figure 8:**
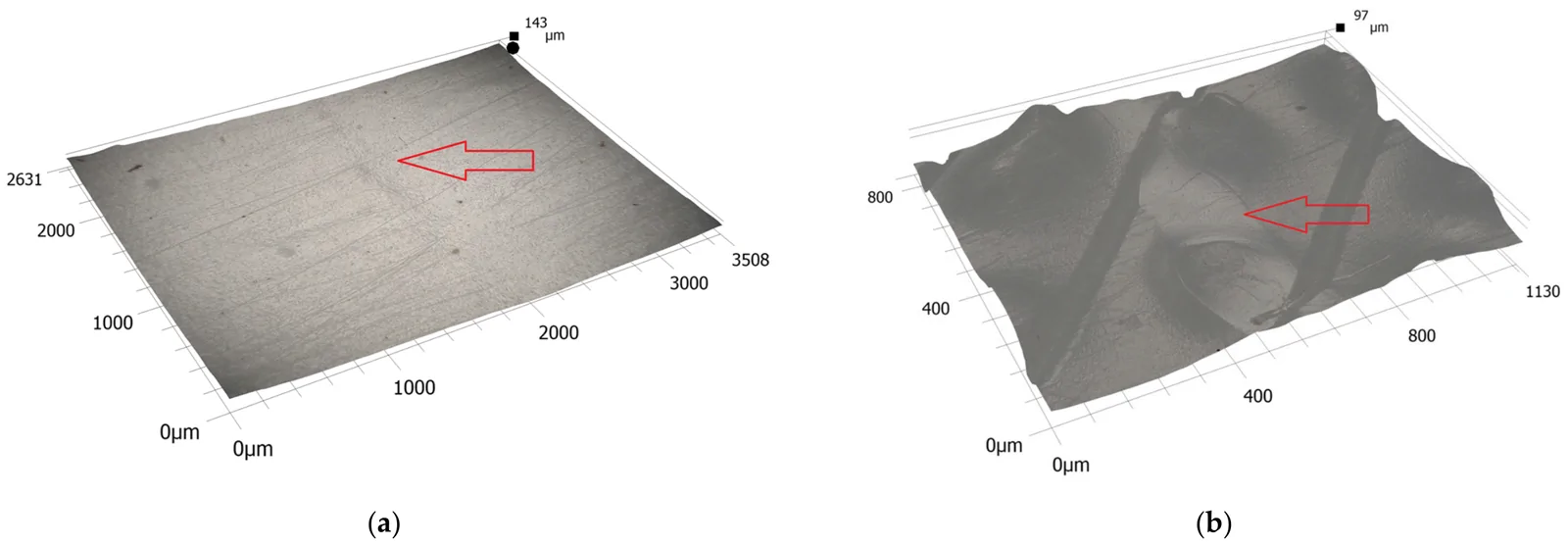
Representative surface damage after the tungsten carbide tip hardness test following thermal exposure at 60 °C: (**a**) polyurethane coating and (**b**) ABS-T.

The polyurethane coating required a critical load of 4 N to produce visible surface damage under both testing conditions (Table 9). Although the critical load remained unchanged after thermal exposure, microscopic observations revealed more pronounced scratch formation on the softened coating surface (Figure 8a). Shaily et al. [39] reported scratch hardness values of 2.0–2.4 kg for polyurethane coatings cured at room temperature and 2.4–3.0 kg for coatings cured at elevated temperature. In their study, the coatings were allowed to cure for at least 24 h, whereas in the present work, the coating was evaluated immediately after thermal exposure. Consequently, the measured behaviour reflects the mechanical performance of the coating at an elevated temperature rather than the effect of additional curing.

Among the 3D-printed polymer specimens, ABS-T exhibited the highest scratch resistance (8 N), followed by PLA and PET-G (6 N). Exposure to 60 °C reduced the critical load only for PLA (6 N → 4 N), whereas ABS-T and PET-G retained their original resistance (Table 9). These results indicate that PLA became more susceptible to surface scratching at an elevated temperature, while ABS-T maintained the highest resistance among the tested polymer materials.

Previous studies have reported different hardness rankings depending on the testing method. Kujawa and Ptak [52] measured Vickers microhardness and found comparable hardness values for ABS and PLA, whereas PET-G showed only slightly lower values. In contrast, Ekrem and Yılmaz [53] reported the highest Shore hardness for PET-G, followed by PLA and ABS. Mishra et al. [43] measured a Shore D hardness of 74.4 for PET-G. Despite these differences, both hardness methods used in the present study consistently identified ABS-T as the material with the highest surface hardness, whereas PLA exhibited the greatest reduction in hardness immediately after thermal exposure.

Microscopic observations further demonstrated that even very narrow scratches produced by the tungsten carbide stylus resulted in permanent surface damage on both the polyurethane coating and the 3D-printed polymer specimens. The higher sensitivity of microscopic evaluation confirmed that surface damage was more extensive than could be detected by visual inspection alone.

The superior hardness of ABS-T is consistent with the findings of Seyedzavvar and Boğa [37] and Isaac et al. [54], who reported that ABS-based materials are well suited for functional and engineering applications because of their favourable combination of mechanical strength and thermal resistance. PET-G, although recognized for its high ductility and impact resistance, exhibits lower thermal resistance and softens more readily at elevated temperatures, which may limit its application in components exposed to increased service temperatures [37,55].

## 4. Conclusions

This study evaluated the effect of immediate exposure to 60 °C on the surface mechanical performance of a conventional polyurethane coating and visible 3D-printed polymer components intended for furniture applications. The specimens were tested immediately after removal from the laboratory dryer, while their surfaces remained at an elevated temperature, enabling assessment of their short-term mechanical response under elevated service-temperature conditions.

The main findings can be summarized as follows:

- Dimensional stability: No measurable dimensional changes or deformation were observed for the polyurethane coating, ABS-T or PET-G, whereas PLA showed slight dimensional changes.
- Mechanical and surface performance: ABS-T exhibited the most balanced performance in terms of impact resistance, abrasion resistance and surface hardness. PET-G showed good dimensional stability and retained its pencil hardness after exposure, but exhibited increased susceptibility to impact cracking. PLA showed slight dimensional changes and earlier crack initiation under some impact conditions, although its abrasion resistance was not reduced by exposure to 60 °C. The polyurethane coating showed the smallest impact indentation but lower abrasion resistance and reduced pencil hardness after exposure.
- Microscopic analysis: Microscopic examination revealed surface defects that were not always detectable by visual inspection. Cracks in the 3D-printed specimens were frequently observed along the deposition pattern of adjacent filaments; this observation suggests that filament interfaces may act as preferential crack-propagation paths.
- Practical implications: ABS-T appears to be the most suitable of the investigated materials for visible furniture components exposed to short-term elevated temperatures, while PET-G should be used with caution in applications exposed to radiant heat or direct sunlight.
- Limitations: The study was limited to short-term exposure at 60 °C and to the investigated materials, specimen geometries and mechanical tests. Therefore, the results should not be directly extrapolated to long-term thermal ageing, other temperatures, or the structural strength of complete furniture joints. Further studies should evaluate longer exposure periods, repeated thermal cycles and different loading conditions.

Overall, the results demonstrate that immediate elevated-temperature exposure can alter the surface mechanical performance of polymer materials and should be considered when selecting materials for visible furniture applications.

## Figures and Tables

**Table 1:** Experimental factors and response variables considered in the study.

Category	Variable	Standard/Method
Experimental factors	Surface finish	PU coating, PLA, ABS-T, PET-G
	Exposure	20 °C, 60 °C for 1 h
Response variables	Impact resistance	STN EN ISO 6272-2 [29]/falling-weight impact test
	Abrasion resistance	STN EN ISO 7784-1 [30]/abrasion test
	Pencil hardness	STN EN ISO 15184 [31]/pencil test
	Scratch resistance	STN EN 438-2+A1 [32]/tungsten carbide tip scratch test

**Table 2:** Polyurethane coating and application parameters.

Finish Product	PU White Satin Top Coat
Commercial name	6OPP530NI
Components	Added UV filters
Hardener (mixing ratio by weight)	6CTH3 (2:1)
Gloss	Matt
Density (WPG)	1.350 ± 0.012 g·cm^−3^ (at 20 °C)
VOC material (g·L^−1^)	445.50
Weight solids (%)	67 ± 1
Application system	Traditional spray gun
Application amount (g·m^−2^)/Coat number	100–150/2
Dry to scuff sand (min)	30–45
Intermediate grinding	P240
Stackable (h)	12 (minimum)

**Table 3:** Printing parameters used for 3D printing of the test specimens.

Specimens	Material	Infill Structure	No. of Layers T/I/B	Layer Height (mm)	Density T/I/B (%)	Flow Rate (%)	Build Plate Temperature (°C)	Printing Speed T/I/B (mm·s^−1^)
I	PLA	Cubic	3/64/3	0.2	100/30/100	0.98	55	270/105/270 270/105/270 135/53/135
	ABS-T					0.95	90	
	PET-G					0.95	80	
II	PLA	None	4/–/1	0.2	100/-/100	0.98	55	270/-/270 270/-/270 135/-/135
	ABS-T					0.95	90	
	PET-G					0.95	80	
III	PLA ABS-T PET-G	None	4/–/1	0.2	100/-/100	0.98 0.95 0.95	55 90 80	270/-/270 270/-/270 135/-/135

Note: T—top (face) layers; I—infill (inner); B—back layers.

**Table 4:** Classification of surface damage after impact testing.

Degree	Visual Evaluation
1	No visible changes
2	No cracks on the surface and the intrusion was only slightly visible
3	Visible light cracks on the surface, typically one to two circular cracks around the intrusion
4	Visible large cracks at the intrusion
5	Visible cracks were also off-site of intrusion, peeling of the coating

**Table 5:** Pencil hardness scale used for surface hardness evaluation.

Pencil Number	1	2	3	4	5	6	7	8	9	10	11	12	13
Pencil Hardness	3B	2B	B	HB	F	H	3H	4H	5H	6H	7H	8H	9H

**Table 6:** Surface temperature after exposure to 60 °C for 1 h and permanent dimensional changes in the tested specimens.

Materials	Specimens	Surface Temperature (°C)	Dimensions (mm)	
			Before Exposure	After Testing
PU coating		59 ± 1/35 ± 1	20.09 × 200.23 × 400.32	20.05 × 200.19 × 400.28
PLA	I	58 ± 1/25 ± 1	79.85 × 119.95 × 14.10	76.14 × 113.14 ×15.22
ABS-T			79.36 × 119.31 × 13.97	79.47 × 119.33 × 14.06
PET-G			79.50 × 119.55 × 13.94	79.61 × 119.36 × 14.01
PLA	II	57 ± 1/27 ± 1	79.21 × 118.92 × 1.04	74.14 × 111.35 × 1.26
ABS-T			78.81 × 118.74 × 0.99	78.80 × 118.61 × 1.01
PET-G			79.12 × 119.07 × 1.04	79.14 × 119.16 × 1.01
PU coating		59 ± 1/38 ± 1	20.09 × 100.18 × 100.24	20.05 × 100.11 × 100.17
PLA	III	57 ± 1/27 ± 1	Ø 99.27 × 1.03	Ø 93.26 × 1.14
ABS-T			Ø 98.35 × 0.98	Ø 98.58 × 1.01
PET-G			Ø 99.03 × 1.02	Ø 97.49 × 1.03

**Table 7:** Evaluation of impact resistance at different drop heights.

Material	Test Condition *	Degree Evaluation					
		Visual Inspection/Digital Microscopy					
		Drop Height (mm)					
		10	25	50	100	200	400
PU coating	20 °C	1/1	1/1	2/4	3/4	4/5	5/5
	60 °C	1/1	1/1	2/4	3/4	4/5	5/5
PLA	20 °C	1/1	1/1	2/2	2/3	3/4	5/5
	60 °C	1/1	1/1	2/2	2/2	3/3	5/5
ABS-T	20 °C	1/1	1/1	2/2	2/2	2/2	4/5
	60 °C	1/1	1/1	2/2	2/2	3/4	4/5
PET-G	20 °C	1/1	1/1	2/2	2/3	3/5	4/5
	60 °C	1/1	1/1	2/3	2/3	3/5	4/5

**Note:** * 20 °C—specimens tested after conditioning under laboratory conditions (20 ± 2 °C and 50 ± 5% RH); 60 °C—specimens tested immediately after 1 h of thermal exposure at 60 ± 2 °C.

**Table 8:** Three-way ANOVA for impact resistance and two-way ANOVA for abrasion resistance.

Factor	Level of Significance—*p*	
	Impact Resistance (mm)	Abrasion Resistance (g)
Material	0.000	0.000
Temperature	0.000	0.000
Drop Height	0.000	–
Material–Temperature	0.000	0.212
Material–Drop Height	0.000	–
Temperature–Drop Height	0.000	–
Material–Temperature–Drop Height	0.000	–

**Table 9:** Surface hardness determined by the pencil test and the tungsten carbide tip test.

Material	Test Condition	Pencil Hardness	Critical Load, Tungsten Carbide Tip (N)
PU coating	20 °C	3H	4
	60 °C	H	4
PLA	20 °C	H	6
	60 °C	F	4
ABS-T	20 °C	4H	8
	60 °C	H	8
PET-G	20 °C	H	6
	60 °C	H	6

**Note:** 20 °C—specimens tested after conditioning under laboratory conditions (20 ± 2 °C and 50 ± 5% RH) and 60 °C—specimens tested immediately after exposure to 60 ± 2 °C for 1 h.

## Data Availability

Data are contained within the article. Further inquiries can be directed to the corresponding author.
